# Ciphers and Executioners: How 3′-Untranslated Regions Determine the Fate of Messenger RNAs

**DOI:** 10.3389/fgene.2019.00006

**Published:** 2019-01-24

**Authors:** Vinay K. Mayya, Thomas F. Duchaine

**Affiliations:** Goodman Cancer Research Centre and Department of Biochemistry, McGill University, Montreal, QC, Canada

**Keywords:** miRNAs, CCR4-NOT complex, RNA binding proteins (RBPs), phase transition, mRNP granules, translational repression, deadenylation, 3′untranslated region (UTR)

## Abstract

The sequences and structures of 3′-untranslated regions (3′UTRs) of messenger RNAs govern their stability, localization, and expression. 3′UTR regulatory elements are recognized by a wide variety of *trans*-acting factors that include microRNAs (miRNAs), their associated machinery, and RNA-binding proteins (RBPs). In turn, these factors instigate common mechanistic strategies to execute the regulatory programs encoded by 3′UTRs. Here, we review classes of factors that recognize 3′UTR regulatory elements and the effector machineries they guide toward mRNAs to dictate their expression and fate. We outline illustrative examples of competitive, cooperative, and coordinated interplay such as mRNA localization and localized translation. We further review the recent advances in the study of mRNP granules and phase transition, and their possible significance for the functions of 3′UTRs. Finally, we highlight some of the most recent strategies aimed at deciphering the complexity of the regulatory codes of 3′UTRs, and identify some of the important remaining challenges.

## Introduction

**Figure 1:**
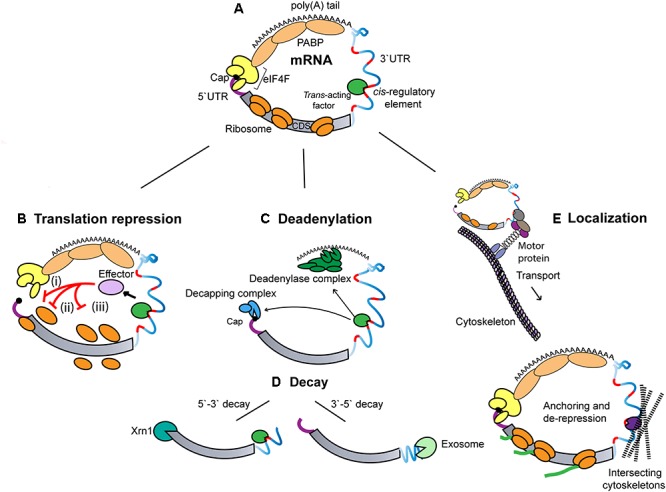
General modes and determinants of 3′UTR in post-transcriptional regulation. **(A)** Schematic illustration of the distinctive features of an eukaryotic mRNA. The 5′-terminal cap structure interacts with the 3′-terminal poly(A) tail of an mRNA through associated eIF4F and PABP. The coding sequence (CDS) is flanked by 5′- and 3′UTR, which harbors *cis*-regulatory sequences (marked in red) and provides a binding platform for *trans*-acting factors (green). **(B)** Translational repression mechanisms. (i) Competition/interference with cap-binding complex, eIF4F (ii) Inhibition of ribosomal subunit joining (iii) Inhibition of translation elongation. **(C)** Deadenylation and decapping. Recruitment of the CCR4-NOT deadenylase complex by *trans*-acting factors catalyzes the deadenylation of the mRNA target. This is often followed by the removal of the 5′-terminal cap structure by the decapping factors (DCP1-DCP2), and the associated co-factors. **(D)** mRNA decay. mRNAs that are deadenylated and decapped are rapidly degraded by either 5′- > 3′ exonuclease (XRN1) or 3′- > 5′ exonuclease (exosome). **(E)** RNA localization. Translationally repressed mRNAs are transported along the cytoskeleton to which it is tethered by RBPs and motor proteins. Upon reaching its destination, the mRNA is anchored, and its translation is de-repressed.

Precise spatial and temporal regulation of gene expression is necessary for the proper development and homeostasis of organisms. Systems approaches indicate that post-transcriptional mechanisms, in particular translational repression is the most significant contributor to establishing a gene’s expression in mammalian cells (Schwanhausser et al., 2011). Post-transcriptional regulation is instated by mechanisms that control translation, stability, and localization of mRNAs. Such mechanisms converge on one or several distinctive features of mRNAs (Figure 1).

The coding sequence (CDS) of an mRNA is flanked by 5′- and 3′-untranslated regions (UTR). These sequences encode regulatory structures and sequences often referred to as *cis*-regulatory, or *cis*-acting elements. When unrepressed, interactions between the 5′-terminal cap, the eIF4F cap-binding complex (an assembly of eIF4E, eIF4A, and eIF4G), the 3′-terminal poly(A) tail and the associated poly(A) binding proteins (PABPs) lead to circularization of an mRNA (Gallie, 1991; Wells et al., 1998). mRNA circularization is thought to allow for synergy of the 5′-cap and poly(A) tail in potentiating translation initiation, and possibly also in stabilizing the mRNA (Sachs et al., 1997; Schwartz and Parker, 1999). Circularization brings 3′UTR *cis*-acting elements closer to the translation initiation machinery. Perhaps not surprisingly, 3′UTR-driven mechanisms determine the expression and fate of mRNAs by targeting the 5′-cap and 3′-poly(A) tail moieties and/or their associated cofactors.

The functional information encoded in the sequence and structure of 3′UTRs are decrypted and acted upon by an array of cellular regulatory factors (often referred to as *trans*-acting factors). Regulatory factors can be broken down into two distinct categories based on their direct molecular implication in (i) *specific recognition* of the 3′UTR sequence and structure, and (ii) *execution* of consequent activities. Factors involved in specific recognition include a variety of non-coding RNAs, such as microRNAs (miRNAs), and RNA-binding proteins (RBPs) to match the sequences and structural determinants encoded in 3′UTRs. A more limited diversity of effector machineries can be grouped in three effector activities: (i) translational control (Figure 1B), most often acting on translation initiation (Nelson et al., 2004; Humphreys et al., 2005; Chendrimada et al., 2007; Mathonnet et al., 2007; Zdanowicz et al., 2009), but also in some cases on translation elongation (Petersen et al., 2006; Gu et al., 2009), (ii) deadenylation and decay (Figures 1C,D), whereby deadenylation of an mRNA can be coupled to some degree to its decapping and decay, and (iii) localization (Figure 1E), which can be established through active RNA transport along the cytoskeleton and/or asymmetric anchoring of an mRNA in a cellular domain.

In many cases, including the examples presented below, more than one effector activity can be mobilized by a 3′UTR. Recognition and effector activities can involve synergistic, cooperative, or coordinated interactions dictated by the 3′UTR regulatory sequences themselves, but also by the cellular, sub-cellular, and biochemical context wherein the mRNA is found. mRNAs and the regulatory machineries are deeply affected by concentration, stoichiometry, affinities, RNA editing, protein post-translational modifications, and physical seclusion, all of which can change with cell identity or adaptation to environmental cues. Directly speaking to both cellular and biochemical contexts and re-emerging with the refining of different classes of RNA-protein condensates (referred to as mRNP granules) is the concept of phase transition. It remains less than clear how phase transition functionally intersects with 3′UTR regulatory mechanisms. Several hypotheses have recently been substantiated and will be discussed later in this review.

## RNA-Binding Proteins (RBPs)

The human genome encodes more than 1,500 RBPs (reviewed in Hentze et al., 2018). Each one of these proteins is constituted of one or more RNA binding domains (RBD), which can be grouped in RBP families, and auxiliary domains that enable other interactions or carry out enzymatic activities (Gerstberger et al., 2014). Canonical RBDs that are often involved in 3′UTR recognition include RNA recognition motifs (RRM), K-Homology (KH) domain, several types of zinc finger domains, double-stranded RNA binding domain (dsRBD), Piwi/Argonaute/Zwille (PAZ) domain, Pumilio/FBF (PUF) domain, and Trim-NHL domain proteins (Lunde et al., 2007). Using intra-molecular or extra-molecular combinations of RBDs, RBPs can improve RNA recognition specificity, affinity, and avidity. Distinct surfaces of RBDs, specific motifs and auxiliary domains mediate the protein-protein interactions required to recruit and activate effector activities to mRNAs.

We will next review some well-characterized examples of how RBPs achieve these functions. Note that RBPs can also play a disruptive role on the activities guided by other regulatory elements in 3′UTRs. Those will be discussed later in this review.

### PUF Proteins

Eukaryotic Pumilio and FEM-3 binding factor (PUF) proteins are part of a family of RBPs that can instigate translational repression, deadenylation and decay of targeted mRNAs. PUF proteins regulate a large number of mRNA targets involved in diverse biological functions. For example, *Drosophila* and *Caenorhabditis elegans* PUF proteins are important for the maintenance of stem cells (Wickens et al., 2002) and target mRNAs of central components of the Ras/MAPK, PI3K/Akt, NF-κB, and Notch signaling pathways (Kershner and Kimble, 2010). In mammalian cells, the precise dosage of PUF proteins is essential to fine-tune the expression of mRNAs encoding mitosis, DNA damage and DNA replication factors. Recently, PUF proteins were shown to be involved in a network of interactions with the NORAD lncRNA at its center, which prevents chromosomal instability (CIN) (Lee et al., 2016).

The PUF family of proteins binds RNAs bearing the 5′-UGUR (where *R* = purine) sequence (Quenault et al., 2011). The determinants of those interactions are understood to such an extent that a PUF protein’s specificity can actually be predicted (Hall, 2016). For example, the classical *Drosophila* Pumilio protein uses its eight α-helical Pumilio repeats to bind the eight-nucleotide sequence 5′-UGUANAUA. Furthermore, Pumilio proteins can be co-expressed. In *Saccharomyces cerevisiae*, co-expression of PUF proteins at different concentrations and with distinct binding affinities can result in competition for individual binding sites (Lapointe et al., 2015, 2017). Binding of PUF proteins to an mRNA typically leads to translational repression, deadenylation, and mRNA decapping. The yeast PUF-domain Mpt5p protein directly interacts with the ortholog of CAF1, one of the two catalytic subunits of the Carbon Catabolite Repressor-Negative on TATA (CCR4-NOT) deadenylase complex, through its RNA-binding domain (Goldstrohm et al., 2006). This interaction is conserved in metazoa, and *C. elegans* and human PUF homologs can also bind to the yeast CAF1 ortholog (Suh et al., 2009; Van Etten et al., 2012; Weidmann et al., 2014). PUF proteins can also repress mRNA expression by inducing their destabilization. Indeed, Mpt5p can recruit an eukaryotic translation initiation factor 4E (eIF4E)- binding protein to target mRNAs (Blewett and Goldstrohm, 2012). eIF4E-binding proteins block the interaction between eIF4E and eIF4G, and this typically prevents the recruitment of the 43S pre-initiation complex (PIC) to mRNAs (Haghighat et al., 1995). However, sometimes including this case, the interaction leads to the recruitment and activation of decapping and decay co-factors (Ferraiuolo et al., 2005; Nishimura et al., 2015).

### Nanos and TRIM-NHL Proteins

The outcome of PUF protein binding to mRNA targets can be altered through interactions with other RBPs. This is the case for the prototypical Pumilio protein in the regulation of *hunchback* mRNA in *Drosophila* (Sonoda and Wharton, 2001), wherein its functions are highly dependent on Nanos and Brain Tumor (Brat) proteins. The RNA-binding specificity of Nanos is defined by its interactions with Pumilio, and Nanos directly interacts with the CCR4-NOT deadenylase complex to promote deadenylation of mRNAs (Curtis et al., 1997; Kraemer et al., 1999; Sonoda and Wharton, 1999; Kadyrova et al., 2007). Brat, a member of the broadly conserved TRIM-NHL family of proteins, forms a ternary complex with Pumilio and Nanos. This complex recruits the effector protein 4EHP to repress the translation of mRNAs (Cho et al., 2006). 4EHP is an eIF4E-like cap binding protein that does not interact with eIF4G and impairs ribosome recruitment to the mRNA (Rom et al., 1998). Unlike Nanos, Brat can stably bind RNA on its own through its NHL domain, and can also function independently of PUF proteins (Laver et al., 2015). Proteomic analysis of CCR4-NOT complex also suggests an interaction with Brat (Temme et al., 2010). It remains unknown whether this is a direct interaction and whether it contributes to and/or is necessary for mRNA repression. TRIM-NHL proteins exert a broader set of biological functions beyond their interplay with Pumilio in *Drosophila* embryo. They play critical roles in brain development, cell polarity, and sex determination (Tocchini and Ciosk, 2015). It is quite possible that this family drives different mechanisms in different cellular or physiological contexts, and that functional interactions with other RBP families may depend on the mRNA target and/or its genetic niche.

### HuR and TTP Proteins

The presence of adenylate/uridylate (AU)-rich sequences in 3′UTRs has long been associated with regulation of mRNA stability (Barreau et al., 2005). Early computational analysis of human mRNA datasets estimated that 8% of mRNAs harbor AU-rich elements (Bakheet et al., 2006). While AU-rich sequences may be expected to contribute to the destabilization of 3′UTR folding structures, they are also directly recognized by a diversity of RBPs. Tristetraprolin (TTP) and its paralogs: butyrate response factors 1 and 2 (BRF-1/2), bind to AU-rich elements through their two zinc-finger domains and promote the decay of mRNAs (Lai et al., 2000). Here again, TTP or BRF direct mRNA destabilization by recruiting effectors of deadenylation, decapping, and 5′- and 3′-exonuclease activities (Lykke-Andersen and Wagner, 2005; Sandler et al., 2011). Interactions with effectors have been mapped to an auxiliary N-terminal domain, which is sufficient to trigger the decay of target mRNAs (Lykke-Andersen and Wagner, 2005). The XRN1 5′- > 3′ exonuclease is thought to be the enzyme effecting mRNA degradation instigated by TTP. It is recruited through the Enhancer of Decapping-4 (EDC4) scaffolding protein (Chang et al., 2014).

Not all AU-rich encoding mRNAs are subjected to degradation. In fact, closely similar sequences can instead lead to enhanced mRNA stability. Such a response often occurs when the HuR protein associates with AU-rich sequences (Brennan and Steitz, 2001). HuR is ubiquitously expressed and belongs to the Embryonic lethal abnormal vision (ELAV) family of proteins (Ma et al., 1996). The exact molecular mechanism used by HuR to confer mRNA stability is still being resolved (von Roretz et al., 2011). An early study showed that overexpression of HuR could slow the decay of mRNAs without impacting their deadenylation rates (Peng et al., 1998). The prevailing model proposes that HuR can stabilize AU-rich encoding mRNAs through competition for binding with factors such as TTP or a subset of miRNAs. Some of the keys to predicting whether an AU-rich sequence dictates degradation, stabilization or has no impact on an mRNA will likely lie in quantitative parameters such as stoichiometry of AU-rich elements and RBPs, and their binding affinities. Future studies may thus benefit from quantitative approaches in specific cell types.

## microRNAs (miRNAs)

miRNAs are genome-encoded, ∼22-nucleotide (nt)-long RNA molecules which guide the associated proteins toward binding sites located in the 3′UTRs of mRNAs to repress their expression. miRNAs were first discovered in *C. elegans* where they regulate the heterochronic cascade of genes that pre-determines cell fate and developmental transitions (the *lin-4* and *let-7* miRNAs) (Lee et al., 1993; Wightman et al., 1993; Reinhart et al., 2000). A turning point for the fields of miRNAs and 3′UTRs was the identification of several *let-7* homologs in other species including humans (Pasquinelli et al., 2000). This discovery coincided with important advances in sequencing technologies and sparked a concerted effort of miRNA sequencing and prediction, leading to the identification of thousands of new miRNAs (Lee and Ambros, 2001; Lau et al., 2001; Lagos-Quintana et al., 2001; Friedman et al., 2009). Currently, more than two thousand miRNAs have been identified in the human genome, and the miRbase database contains 48,885 mature miRNAs from a total of 271 species (Kozomara and Griffiths-Jones, 2014). Since their conservation across species has been shown, miRNAs have been implicated in a myriad of functional cascades across metazoans, including development, signaling, immune system, and metabolism (Ameres and Zamore, 2013). Conversely, their mis-expression or misregulation contributes to or plays instrumental roles in a variety of diseases ranging from heart disease to diabetes to cancer (Hesse and Arenz, 2014).

The base-pairing of miRNAs with 3′UTR sequences is quite distinct from what is to be expected from a ‘free’ single-stranded RNA of the same length. A miRNA’s target recognition kinetics and specificity are largely dictated by its interactions with the Argonaute protein within which it is bound in the cell (for a review, see Duchaine and Fabian, 2018). The miRNA strand is stretched across Argonaute’s croissant-shaped structure by interactions with its four domains. On its 5′ end, the miRNA interacts with the Mid and PIWI domains. Across a central cleft, the 3′ end of the miRNA is bound to the PAZ domain which closely interacts with the N-domain. Extensive interactions pre-orients the 5′-most bases of the miRNA (nts 2-8), a region called the seed, into a favorable conformation for pairing with target sequences. Target recognition through the seed is a two-step process wherein the rate limiting step is the pairing of nts 2–5 and the dissociation rate is largely determined by the pairing of nts 6–8 (Wee et al., 2012; Schirle et al., 2014; Chandradoss et al., 2015; Salomon et al., 2016). Multiple genomic studies and individual miRNA-binding sites have indicated that, alternative non-canonical routes of target recognition may be prevalent. For example, some miRNAs further use the 3′ end of the miRNA in target recognition (Broughton et al., 2016; Brancati and Großhans, 2018). Such alternative modes of target recognition likely involve dynamic interactions with the N-PAZ pair of Argonaute domains.

The importance of the interactions and molecular mechanics of the Argonaute scaffold in dictating miRNA targeting kinetics recently led the Zamore group to suggest that the miRNA/Argonaute (a minimal assembly referred to as RISC) behaves as a ‘programmable RNA-binding protein’ (Salomon et al., 2016). Incidentally, this analogy further extends to the effector activities that are mobilized by miRNAs, which largely overlap with effectors and mechanisms mobilized by RBPs. Metazoan Argonautes that are programmed by miRNAs also stably interact with the TNRC6 or GW182 family of proteins. This constitutes the core of a complex often referred to as miRNA Induced Silencing Complex or miRISC (Jonas and Izaurralde, 2015). In essence, GW182 proteins bridge interactions between Argonaute proteins and effector complexes including mRNA deadenylation, decapping and decay machineries. Here again, the CCR4-NOT complex plays a central and pivotal role (Fabian et al., 2011; Braun et al., 2013; Jonas and Izaurralde, 2015). We will thus next examine in more details the architecture, interactions and important functions of the CCR4-NOT complex in determining the fate of mRNAs.

## The CCR4-NOT Complex: A Hub for 3′UTR Effector Activities

**Figure 2:**
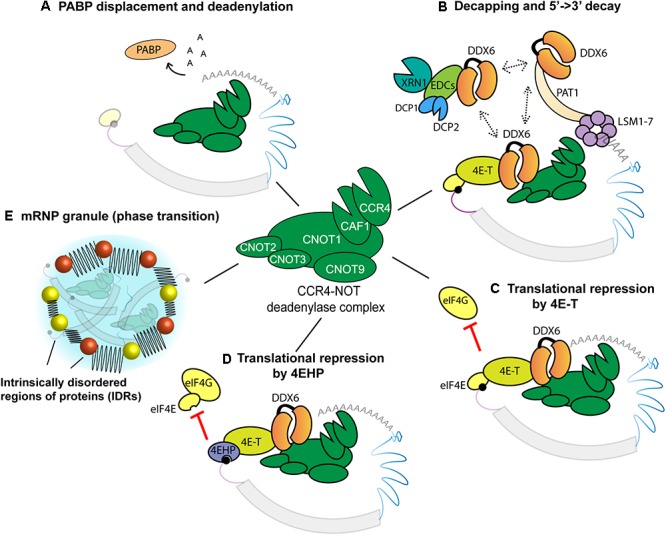
Roles of the CCR4-NOT complex and associated proteins in effecting 3′UTR-encoded gene regulation. The CCR4-NOT complex contains at least eight subunits, of which only six are shown here. **(A)** Inhibition of mRNA circularization by PABP displacement and deadenylation. **(B)** CCR4-NOT-directed mRNA decay. The CCR4-NOT complex deadenylates the mRNA and recruits DDX6. DDX6 promotes decay through three mutually exclusive interactions, with 4E-T, EDC-3, and PAT1 (dashed lines). **(C,D)** Inhibition of translation through CCR4-NOT. Note that all mechanisms depicted target initiation. **(C)** DDX6 recruits 4E-T to prevent the binding of eIF4G to eIF4E. **(D)** 4EHP is recruited to the cap through 4E-T/DDX6/CCR4-NOT complex. **(E)** Assembly of an mRNP granule. CCR4-NOT and intrinsically disordered regions (IDRs)-encoding proteins are sequentially recruited to target mRNAs to promote mRNP formation, possibly enabling or promoting phase transition.

The CCR4-NOT complex plays a central role in the fate of an important diversity of mRNAs. Other deadenylases such as the PAN2/3 complex exert a regulatory function, but on a more limited subset of mRNAs and on population of longer poly(A) tails (Chen and Shyu, 2011). However, the CCR4-NOT complex seems to be responsible for most poly(A) tail controls in metazoan transcriptomes where it has been examined (Tucker et al., 2001; Temme et al., 2004; Yamashita et al., 2005; Schwede et al., 2008; Nousch et al., 2013). The CCR4-NOT complex integrates the effector functions in mechanisms initiated by a diversity of RNA-binding proteins and miRNAs (Figure 2). CCR4-NOT consists of two highly conserved modules: the CNOT1/2/3 proteins constitute a scaffolding module for all the subunits of the complex, while the catalytic module of the complex is formed by two deadenylases, EEP-type CCR4 and DEDD-type CAF1. Their functions partially overlap or compensate for each other *in vivo*, but CAF1 is believed to assume the bulk of the function in miRNA-directed deadenylation (Fabian et al., 2009). Beyond scaffolding the CCR4-NOT complex, the central CNOT1 subunit acts as a tether and directly interacts with GW182, TTP, Nanos, PUF, Smaug, and several other RNA-binding proteins in different cells and organisms (Wahle and Winkler, 2013).

Recruitment of the CCR4-NOT complex to mRNAs is associated with its deadenylation activities, but a different perspective on the function of this complex has recently emerged. The CCR4-NOT complex also recruits distinct activities such as decapping and exonucleases (Figure 2B) that are often coupled with deadenylation, but also with cap-binding and translation repression without mRNA deadenylation or decay (Figures 2C,D). Its interactions with intrinsically-disordered region (IDR)-encoding proteins that are components of the mRNP in the *C. elegans* embryo recently suggested a role in nucleating phase transition (Wu et al., 2017) (Figure 2E).

### mRNA Deadenylation and Decay

In addition to its role in translation initiation, PABP is a cofactor of deadenylases, including the CCR4-NOT complex (Fabian et al., 2009; Huntzinger et al., 2013). *In vitro*, PABP accelerates the deadenylation of long 3′UTRs for which the poly(A) tail is distant to the regulatory sequences (Flamand et al., 2016). The first step in deadenylation of an mRNA is thought to be the displacement of PABP proteins from the poly(A) tail by cofactors recruited through the GW182 protein and CCR4-NOT complex (Moretti et al., 2012; Zekri et al., 2013). Removal of the poly(A) tail is then catalyzed by the CAF-1 and CCR4 deadenylases subunits (Figure 2A).

In metazoans, deadenylation is often tightly coupled with mRNA decapping and decay (Figure 2B). Earlier studies showed that following the shortening of a poly(A) tail below a certain threshold, an mRNA is subjected to first-order decay (Chen et al., 2008). mRNA deadenylation and decay are clearly coupled in early zebrafish embryo, where mRNA deadenylation instigated by the miR-430 family of miRNAs marks the initial step in the decay of an important fraction of maternal mRNAs in the Maternal-to-Zygotic Transition (MZT) (Giraldez et al., 2005, 2006). This is also obvious in *Drosophila* S2 cultured cells, where fully deadenylated mRNAs do not accumulate, and impairing the decapping enzymes Dcp1/2 is necessary to detect the deadenylated species (Eulalio et al., 2009). The LSM1-7 proteins are thought to form a ring-like complex around the remnants of the shortened poly(A) tail and to promote mRNA decapping and decay (Tharun, 2009).

A key protein, which physically couples the CCR4-NOT complex with decapping and decay, is the DEAD-box protein DDX6. DDX6 directly interacts with CNOT1 subunit and multiple decapping/decay factors, either simultaneously or through mutually exclusive interactions (Tritschler et al., 2009; Sharif et al., 2013; Chen et al., 2014; Mathys et al., 2014; Rouya et al., 2014; Nishimura et al., 2015; Ozgur et al., 2015). Interestingly, DDX6 also interacts with eIF4E-transporter (4E-T). This interaction is thought to increase the local concentration of decapping factors such as DCP2 around the 5′-cap, thus enabling competition with eIF4E (Nishimura et al., 2015). The removal of the 5′-cap structure by DCP2 seals the fate of the mRNA toward degradation via the 5′- > 3′ decay pathway mediated by XRN1 (Arribas-Layton et al., 2013). The activity of DCP2 is greatly enhanced by DCP1 and additional factors such as enhancers of decapping (EDC-3, EDC-4), PAT1, and the LSM1-7 complex (Jonas and Izaurralde, 2013) (Figure 2B). Alternative routes of mRNA decay have also been proposed, which would proceed from the 3′ end and through the cytoplasmic exosome complex (Chen and Shyu, 2011).

### Translational Repression

mRNA deadenylation abolishes the physical and functional synergy between the 5′-cap and poly(A) tail, resulting in translational repression (Mishima et al., 2006; Wakiyama et al., 2007). However, strong evidence indicates that the CCR4-NOT complex can also participate in direct translational repression, through mechanisms that do not involve its deadenylase activities (Figures 2C,D). Using luciferase reporters engineered to block deadenylation, an early study showed that tethering of *Xenopus* or human CAF1 is sufficient to repress mRNAs (Cooke et al., 2010). Several other reports, using different experimental designs and systems, have since then confirmed the role of CCR4-NOT as a direct translational repressor (Braun et al., 2011; Chekulaeva et al., 2011; Flamand et al., 2016; Chapat et al., 2017). Models proposed to explain this activity have accumulated in recent years and were substantiated to different extents. Disruption of mRNA circularization by displacement of PABP through CCR4-NOT and its cofactors has been suggested as one mechanism (Zekri et al., 2013). Other mechanisms instead revolve around displacement of interactions with the 5′-cap of targeted mRNAs, and DDX6 is also central for these functions of CCR4-NOT.

DDX6 can recruit 4E-T whose interaction with eIF4E can displace eIF4G and thus mediate translational repression (Kamenska et al., 2016). Repression can also occur through the strong interaction between 4E-T and 4EHP (Joshi et al., 2004; Cho et al., 2005). Recruitment of this dimer to CCR4-NOT through DDX6 was recently involved in translation repression by miRNAs (Chapat et al., 2017). A subset of mRNAs is translationally regulated through this 4EHP-4E-T mechanism in mammalian cells, among which DUSP6 plays an important role in fine-tuning the ERK signaling cascade (Jafarnejad et al., 2018). This last study is unique in identifying a physiological purpose to one of the many CCR4-NOT ‘pure’ translational repression mechanisms. Indeed, the physiological importance has yet to be determined for most of those mechanisms, which were identified in cell culture and/or *in vitro*. It remains possible that distinct mechanisms will be predominant in different cellular contexts or on particular mRNA targets.

## Cooperative and Competitive Interplay Among RBPs and miRISC

RBPs and miRISC can interact among themselves and with each other to alter the fate of mRNAs through either cooperation or competition. Considering the importance of 3′UTR sequences and the diversity and density of potential binding sites for RBPs and miRNAs, it is hard to expect otherwise. The median length of human 3′UTRs is 1,200 nt (Jan et al., 2011). On average each mRNA 3′UTR is bound by 14 RBPs (Plass et al., 2017), and ∼70% of vertebrate 3′UTRs encode multiple sites for different miRNA families (Friedman et al., 2009). Neither miRNA- nor RBP binding sites are distributed randomly in 3′UTR sequences. Early on, genomic studies have shown that miRNA-binding sites are more likely to be functional when they are located close to each other, or when located close to the ORF or the poly(A) tail (Grimson et al., 2007; Saetrom et al., 2007). Similarly, genomic analyses indicate that AU-rich sequences are associated with a greater functional output of nearby miRNA-binding sites, and computational analyses of the mammalian genomes indicate that recognition sites for PUF proteins and AU-rich sequences are enriched within 50 nt of binding sites for a subset of miRNAs (Jiang et al., 2013).

### miRNA–miRNA Cooperativity

Signs that miRNA-mediated silencing acts through a cooperative mechanism were already visible in the seminal discovery papers in *C. elegans*. The 3′UTR of *lin-14* encodes 7 potential base-pairing sites (Lee et al., 1993), while the *lin-41* 3′UTR harbors two *let-7* miRNA-binding sites, separated by intervening sequences of 27 nt in length (Reinhart et al., 2000). If each of these individual sites were independently functional, some degree of redundancy could be expected, with their individual impairment having limited to no consequence. Instead, both *let-7* sites in the *lin-41* 3′UTR are important *in vivo* (Vella et al., 2004). Likewise, binding sites for *lin-4* and *let-7*, and multiple sites for *lsy-6* functionally interact on the *lin-28* and *cog-1* mRNAs, respectively (Moss et al., 1997; Reinhart et al., 2000; Didiano and Hobert, 2008). *In vitro* and *in vivo* studies later demonstrated that miR-35 and miR-58 miRNAs cooperate in the deadenylation and the silencing of the *C. elegans egl-1/BIM* mRNA (Wu et al., 2010; Sherrard et al., 2017). In addition to the fore-mentioned early genomic studies, which support miRNA cooperativity, mammalian reporter assays clearly confirmed that a combination of sites exert a much more potent silencing output (Broderick et al., 2011). While some studies examined miRNA-binding site cooperativity on natural or fragments of 3′UTR sequences (Koscianska et al., 2015; Schouten et al., 2015), there are few detailed studies of miRNA-binding site interplay.

The mechanisms underlying miRNA cooperativity are still poorly resolved, but three models have been proposed and two have been substantiated experimentally. First, miRISC binding to nearby miRNA-binding sites can enhance their affinity for the 3′UTR (Broderick et al., 2011; Flamand et al., 2017). This type of cooperativity in target binding is in fact required for some non-seed miRNA-binding sites to be stably bound by miRISC and to be functional (Flamand et al., 2017). A second model involves the cooperative recruitment of effector machineries. In an embryonic cell-free system, a reporter mRNA bearing a single miRNA-binding site was not deadenylated, and could not recruit the CCR4-NOT complex, whereas a reporter encoding three adjacent miRNA-binding sites did so efficiently (Flamand et al., 2017). Whether this mode of cooperativity is especially important in the embryo and/or in *C. elegans* is not known at present. A third, mutually not exclusive, possibility could involve the cooperative *activation* of effector activities. CCR4-NOT recruitment by miRISC on 3′UTRs may not be sufficient on its own to trigger mRNA deadenylation and decay. A stoichiometric threshold, a specific configuration of target sites, post-translational modifications and/or conformation changes of miRISC may be required to trigger effector activation. These variations would be consistent with other protein/nucleic acid interaction paradigms, such as transcription factors.

### RBP-miRISC 3′UTR Interactions

RBPs, miRNAs and the associated machineries can regulate their activities through cooperative or competitive interplay. It is likely that the mechanisms at work in cooperating miRNA-binding sites may also explain some of the RBP-miRNA cooperativity. Putative examples of direct interplay may include the cooperation of TTP with miR-16 in regulating TNF-alpha mRNA (Jing et al., 2005), and AU-rich sequences near the miR-16 binding site in the 3′UTR of COX-2 mRNA (Young et al., 2012). Positive interplay can also be indirect, through the modulation of global or local 3′UTR structures. Because they do not code, 3′UTRs can adopt complex folding structures, which can have positive or negative impacts on overlapping or nearby regulatory sequences. Structures can constitute determinants for the recognition of other RBPs, or limit binding to miRNA-binding sites. In turn, binding of miRISC or RBP to high-affinity sites can destabilize folding structures and facilitate access to nearby binding sites. This model explains the effect of Pumilio on the 3′UTR of the p27 tumor suppressor. Pumilio binding promotes a change in the local structure of the RNA that allows the binding of miR-221 and miR-222, leading to silencing of the p27 mRNA (Kedde et al., 2010). Similarly, a study showed that HuR could enhance the activity of *let-7* on c-Myc mRNA. This is also likely through a change in the local structure of the RNA resulting in the unmasking of the *let-7* binding site (Kim et al., 2009).

In the simplest form of antagonistic interaction, overlapping or nearby binding sites can lead to direct competition between RBPs and miRNAs/miRISC through steric hindrance. A survey by Keene and colleagues suggested that HuR prevents the function of abundant miRNAs on nearby and overlapping sites in a subset of mRNAs in HEK293 cells (Mukherjee et al., 2011). Similarly, Fillipowicz and colleagues showed that HuR could displace miRISC bound to a target mRNA thereby alleviating miRNA-mediated repression. This displacement occurs when HuR binds to AU-rich sequences 20–50 nt away from the miRNA-binding site (Kundu et al., 2012), again suggesting steric interference. The HuR example illustrates the fact that an RBP can have both positive or negative impacts on miRNA-binding site function, depending on 3′UTR structure and binding site positioning. It also highlights that interactions between 3′UTR structures, regulatory sequences and their trans-acting factors are precisely tuned through co-evolution.

## Coordinated and Sequential 3′UTR Activities

Beyond simple positive or negative interplay, 3′UTR sequences can lead to the coordination of post-transcriptional mechanisms in both time and space. The mechanism underlying miRNA-mediated silencing is in itself a coordinated series of events wherein mRNA translation repression precedes deadenylation, which in turn precedes decapping and decay. Translation repression can be resolved *in vitro* in a mammalian cell-free system (Mathonnet et al., 2007), *in vivo* in cell culture (Djuranovic et al., 2012), and even occur at distinct but subsequent developmental stages during early zebrafish embryo development (Bazzini et al., 2012). The biological purpose of this series of events, however, remains to be fully elucidated. Some of these steps in the silencing mechanism may be expected to be at least partially redundant with regards to the impact on gene expression. However, one possibility is that translation inhibition enables faster repression, e.g., when a binary decision is promptly required. Another possibility is that this allows for reversible repression in the early steps, whereas decapping and decay may offer a more permanent decision.

### RNA Localization

The coordination of 3′UTR-driven activities is clearly illustrated through examples of active mRNA transport and localization. A majority of mRNAs are localized to subcellular regions and most examples where the underlying mechanisms have been detailed involve 3′UTR regulatory elements (Jansen, 2001; Lécuyer et al., 2007). mRNA localization can be achieved through several mechanisms (reviewed in Martin and Ephrussi, 2009). In active mRNA transport, the mRNA is assembled in a ribonucleoprotein (mRNP) complex through the specific binding of a combination of RBPs to the 3′UTR of an mRNA (Figure 1E). Bound RBPs recruit effector proteins that repress translation and mediate interactions with motor proteins. The repressed mRNP is then transported via the cytoskeleton until it reaches its destination where it is anchored. The mRNA is then de-repressed at the appropriate time and place through a series of events involving displacement/competition by other RBPs, and/or post-translational modifications (reviewed in Besse and Ephrussi, 2008).

### Oskar mRNA Localization

Localization of *oskar* mRNA in the *Drosophila* oocyte is the archetype, and remains one of the best-characterized examples of active mRNA transport (Ephrussi et al., 1991; Kim-Ha et al., 1991). *oskar* mRNA localization to the posterior pole of the oocyte occurs via microtubules through interactions with Staufen (Stau), tropomyosin and EJC components (Micklem et al., 2000; Zimyanin et al., 2008). Localized expression of *oskar* ensures proper patterning of the posterior body axis and germline fate (Kim-Ha et al., 1991). Mislocalization to the anterior pole leads to ectopic formation of abdomen and germ cells (Ephrussi and Lehmann, 1992), and absence of Oskar protein leads to loss of germ cells and aberrant abdominal segments (Lehmann and Nusslein-Volhard, 1986). Moreover, premature translation of localizing *oskar* mRNAs also results in patterning defects (Smith et al., 1992). Translational repression is achieved by Bruno RBP binding to multiple elements in the 3′UTR of *oskar* mRNA (Kim-Ha et al., 1995), which also recruits an eIF4E-binding protein, Cup (Wilhelm et al., 2003) to the mRNA. Similar to the 4E-T protein, Cup disrupts the interaction between eIF4E and eIF4G and prevents 43S pre-initiation complex binding to *oskar* mRNA (Nakamura et al., 2004). Bruno further represses *oskar* mRNA by promoting its oligomerization, a process which likely also contributes to rendering it inaccessible to the translation machinery (Chekulaeva et al., 2006). The Polypyrimidine Tract-Binding protein (PTB), which binds to multiple sites in *oskar* mRNA 3′UTR, is also essential for mRNA oligomerization and densely packed mRNP particles (Besse et al., 2009).

The fates of *oskar* and *nanos* mRNAs are closely linked in the *Drosophila* oocyte. *nanos* mRNA is also localized to the posterior pole, and is rapidly deadenylated and degraded elsewhere in the early embryo through the recruitment of CCR4-NOT complex by Smaug (Smibert et al., 1996, 1999; Bashirullah et al., 1999; Dahanukar et al., 1999; Zaessinger et al., 2006). Translation of *nanos* mRNA at the posterior pole is thought to be activated by the Oskar and Vasa proteins, but the exact underlying mechanism remains unclear (Ephrussi and Lehmann, 1992; Smith et al., 1992). Oskar could inhibit the function of Smaug, either by affecting the binding of Smaug to *nanos* mRNA or by interfering with the recruitment of the CCR4-NOT complex. It is also clear that some of the keys to solving the underlying mechanism will stem from the properties of phase transition in the posterior pole germ plasm (see below).

### mRNA Routes in Mammalian Cells

An important variety of RNA localization events have been described in mammalian cells. Among them, the cascades dictated by the Zipcode and A2RE/RTS *cis*-acting elements provide well-delineated examples of how mammalian mRNAs can be sorted and locally translated in distinct cell types through information encoded in 3′UTRs. They also illustrate how localized cellular signaling can determine the precise site of translation of localized mRNAs.

#### Zipcode and the Zipcode-Binding Protein 1

β-actin mRNA localizes to the leading edge of the fibroblasts (Lawrence and Singer, 1986), and analogous mechanisms are thought to be at work in developing neurites and hippocampal dendrites (Bassell et al., 1998; Zhang et al., 1999, 2001; Eom et al., 2003; Shav-Tal and Singer, 2005). Localization of β-actin mRNA is instigated by the zipcode binding protein 1 (ZBP1) (Ross et al., 1997), which specifically binds a 54-nt long 3′UTR segment termed the ‘Zipcode’ (Kislauskis et al., 1994). The motor for β-actin mRNA localization in fibroblasts was only recently identified (Song et al., 2015). KIF11, a tubulin-associated motor, associates with the β-actin mRNPs wherein it directly interacts with ZBP. Disruption of this interaction *in vivo* leads to β-actin mRNA mis-localization and perturbs cell motility.

The exact nature of the mechanism responsible for the silencing of transported β-actin mRNAs remains unclear. Single-cell live imaging revealed an anti-correlation between the association of ZBP1 or ribosomes with β-actin mRNA (Wu et al., 2015). The authors thus proposed that the packaging of β-actin mRNA into mRNP granules may seclude mRNAs from ribosomes, and thus pre-empt translation. On one hand, the pervasive nature of mRNP granule formation in mRNA localization suggests that translation repression may be at least partly achieved through packaging of such mRNPs. On the other hand, the events leading to localized mRNA translational de-repression are rarely defined. For β-actin mRNA, this appears to result from signaling cascades locally converging on trans-acting factors. Upon reaching the endpoint of mRNA transport, phosphorylation of ZBP1 on a tyrosine residue by the protein kinase Src, which is closely associated with the cell membrane, disrupts RNA binding and relieves β-actin mRNA from translational repression (Hüttelmaier et al., 2005).

#### The A2RE/RTS Pathway

The A2 response element (A2RE) or RNA trafficking signal (RTS) is an 11-nt *cis*-acting element recognized by the heterogenous nuclear ribonucleoprotein A2 (hnRNP A2) and CArG-box binding factor A (CBF-A) proteins. The importance of this element was originally described in the transport of Myelin Basic Protein (MBP) mRNA in oligodendrocyte processes (Ainger et al., 1997; Carson et al., 1997; Hoek et al., 1998; Munro et al., 1999). A2RE/RTS-like sequences have since then been identified in a growing number of localized transcripts including BC1, αCaMKII, NG, ARC, BDNF, *Prm2* mRNAs, and HIV RNAs (Mouland et al., 2001; Muslimov et al., 2006; Gao et al., 2008; Raju et al., 2011; Fukuda et al., 2013). Though the mechanism of translation inhibition remains unclear for most of these mRNAs, assembly of MBP mRNA molecules into granules somehow maintains the transcripts in a repressed state. Just like for β-actin mRNA, phosphorylation of a *trans*-acting factor is key to enable the translation of MBP mRNA, which is released at sites of glia-neuronal contacts through phosphorylation of hnRNP A2 and hnRNP F by the Fyn kinase (White et al., 2008, 2012).

### *Xenopus* Oocyte mRNA Localization Pathways

The developing *Xenopus laevis* oocyte features mRNA localization examples that illustrate how elements in 3′UTRs direct toward distinct localization path in successive stages of development. During the six stages of oogenesis, RNAs localize along the animal/vegetal (A/V) axis of the oocyte (Kloc et al., 2001) through the early and late pathways. In the early pathway, germ plasm RNAs such as DEADSouth, Xpat, Xcat2, and Xdazl are transported by associating with a membrane-less structure termed the mitochondrial cloud (MC) or Balbiani body. This body contains germinal granules, endoplasmic reticulum, mitochondria, and is surrounded with bundles of intermediate filaments, which were suggested to play a role in maintaining its structure (Heasman et al., 1984; Forristall et al., 1995; Kloc and Etkin, 1995; Gard et al., 1997; King et al., 2005; Carotenuto and Tussellino, 2018). During stage II of oogenesis, the mitochondrial cloud expands between the nucleus and vegetal cortex. This expansion is thought to ‘push’ the germinal granules and RNAs toward the vegetal cortex where they are anchored on the cytoskeleton (Alarcon and Elinson, 2001; Wilk et al., 2005). Two distinct localization elements (LE) are encoded in the Xcat2 mRNA 3′UTR (Mosquera et al., 1993). A proximal 240 nt-long element is required for mitochondrial cloud localization (MCLE), whereas a distal ∼160 nt-long germinal granule localization element (GGLE) enables incorporation into germinal granules present inside the MC. Both localization signals are necessary for the proper localization of Xcat2 mRNA, which highlights the coordinated contributions of both 3′UTR elements (Kloc et al., 2000). Xcat2 mRNA is translationally repressed in the MC (MacArthur et al., 1999), and a few studies have implicated the RNA-binding protein Hermes in the repression of Xcat2 mRNP (King et al., 2005; Song et al., 2007; Nijjar and Woodland, 2013).

In the late localization pathway, mRNAs involved in somatic cell fates such as Vg1 and VegT are transported to the vegetal cortex in a microtubule-dependent mechanism (Kloc and Etkin, 1998). The 3′UTR of Vg1 mRNA encodes a 340-nt long LE, wherein clusters of short motifs are bound by the Vera and hnRNP I proteins (Deshler et al., 1997). The Vg1 LE is thought to be initially recognized by hnRNP I. This interaction remodels the Vg1 mRNP, which in turn allows Vera to bind Vg1 mRNA directly. Other factors are then recruited to the Vg1 mRNP including Staufen, Prrp and a kinesin motor to enact localization (Zhao et al., 2001; Yoon and Mowry, 2004; Lewis and Mowry, 2007; Lewis et al., 2008). Only after localizing to the vegetal cortex at the late stage IV of oogenesis is Vg1 mRNA translated (Dale et al., 1989; Tannahill and Melton, 1989). The spatiotemporal control of Vg1 mRNA translation is dictated by the 250-nt long translation-control element (TCE) encoded downstream of the Vg1 LE (Wilhelm et al., 2000; Otero et al., 2001). ElrB, a member of the ELAV family of RBPs, interacts with the TCE of Vg1 mRNA (Colegrove-Otero et al., 2005). This interaction correlates with the repression of the Vg1 mRNA, but how ElrB effects translational repression is not known.

## mRNPs: Going Through Phases in the Lives of mRNAs

Mechanisms involving 3′UTR regulatory elements have long been associated with large mRNP granules. These granules can reach massive sizes by molecular standards (Brangwynne, 2013), often rivaling organelles. The list of large mRNP granules is rapidly expanding and includes P-bodies (originally named GW bodies), germ granules (also called polar granules and P granules, depending on species), stress granules, and the mRNA transport particles (Voronina et al., 2011), among others. Similarities and differences in the composition of large mRNPs have been documented (Eulalio et al., 2007a), mainly through comparison of associated markers by immunofluorescence. For example, stress granules are often distinguished from co-expressed P-bodies through exclusive colocalization of G3BP and DCP2, respectively (Ingelfinger et al., 2002; Tourriere et al., 2003; Kedersha and Anderson, 2007). In the early embryo, germ granules are distinguished from P-bodies through their association with germline markers such as PIE-1 in *C. elegans* (Strome, 2005). The absence of membranes in these organelle-sized particles and their scale led to their non-specific description as ‘large aggregates’ of RNA and proteins. A function in local mRNA concentration or storage for germ granules was naturally inferred from their scale and their concentration of maternal mRNAs in the oocyte (Noble et al., 2008; Voronina et al., 2011). Their importance in storage and protection of subsets of mRNAs from degradation was substantiated by well-defined examples, including the above-described *nanos* mRNA in *Drosophila*. The mRNA storage/protection model for mRNPs is also often associated with seclusion from the translational machinery. For example, in the developing oocytes of *C. elegans*, P granules help store translationally silent transcripts to prevent premature differentiation (Boag et al., 2008). Later in the embryo, P granules selectively repress somatic mRNAs in the P-lineage blastomeres, but not germline mRNAs to maintain germline fate and totipotency (Gallo et al., 2010; Updike et al., 2014).

While a role in mRNA storage makes sense and appears to be well supported, the biochemical nature of large mRNPs has remained elusive since the identification of the electron-dense ‘nuage’ structures in the early days of germline and developmental biology (Wilsch-Bräuninger et al., 1997). A breakthrough was recently made in the mechanisms of assembly and disassembly of mRNP granules. Hyman and colleagues showed that P granules in fact form by phase separation. Granules have liquid-like properties that permit dynamic fusing and exchange of components, but segregate from their surroundings like oil from water (Brangwynne et al., 2009). Similar properties were also described for P-bodies and stress granules *in vitro* (Lin et al., 2015; Molliex et al., 2015). Intrinsically disordered proteins (IDPs) or proteins with at least a portion of disordered regions (IDRs) are a critical component of phase transition and mRNPs (Brangwynne et al., 2015). It is suspected that most, if not all mRNP granules contain different IDPs/IDRs (Uversky, 2017), and the interactions and properties of these proteins can control mRNP contents. Another typical property is their propensity to scaffold multiple proteins through multivalent interaction networks (van der Lee et al., 2014). Alongside IDP/IDRs, mRNAs and their interactions contribute to mRNP dynamics, either in promoting (Lin et al., 2015), or modulating granule assembly (Hubstenberger et al., 2015; Seydoux, 2018). Thus, the nature of protein-protein and protein-RNA interactions which contribute to assembly and stability of mRNP granules are distinct from what is observed in stable complexes in aqueous phases. Phase separation instead is governed by weak multivalent interactions that segregate interacting macromolecules away from water at a critical concentration (Li et al., 2012; Hyman et al., 2014; Banani et al., 2017). Traditional protein-protein interaction studies based on co-immunoprecipitation and *in vitro* interaction assays may not be suitable to detect many, if not most of the interactions that occur in mRNPs. This, in turn, may be one of the reasons why proximity-based interaction mapping methods such as BioID were fruitful in mapping interactions in P-bodies and stress granules (Youn et al., 2018).

In light of the newly discovered properties of mRNPs, new and important questions have emerged. What are the folding and enzymatic differences that prevail in such phase-separated liquid droplets? How is the specific composition (if any) of an mRNP defined, and how are biochemical boundaries maintained or crossed between different types of mRNPs? Earlier work by the Seydoux group revealed that P granules and P-bodies closely interact, but do not merge in the *C. elegans* early embryo (Gallo et al., 2008). More recently, their work identified an important role for IDP MEG-3 in modulating the structural stability of P granules. Different enrichments in PGL-1 and MEG-3 proteins significantly altered mRNP properties and could limit access to RNA (Smith et al., 2016). In the *Drosophila* oocyte, *nanos* mRNPs progress along the cytoskeleton from smaller localization particles to the larger germ granules at the posterior pole. The Gavis group used quantitative single-molecule imaging to analyze the localization dynamics and assembly of mRNP germ granules in the *Drosophila* oocyte. Interestingly, single mRNP complexes that contain individual *nanos* transcripts merge into multi-mRNA granules at the posterior pole. This localized ‘growth’ appears to be exponential, rather than additive, which could be interpreted as mRNPs merging through phase transition into the germ plasm. In contrast, the *oskar* mRNA localizes as multi-copy mRNPs which are segregated from other mRNP granules once it reaches the posterior pole, and this exclusivity contributes to proper germline specification (Little et al., 2015). This suggests that single- or multi-mRNPs, can be differentially transported and locally stored. It further strengthens and refines the links between mRNPs and the transport and localization of mRNA granules.

The possible implications of this mechanism reach far beyond *C. elegans* and *Drosophila* oocytes and embryo. For example, analogous mRNP granules are likely common in mammalian neurons. A study took advantage of the preferential precipitation of IDPs by the chemical biotinylated isoxazole (b-isox) to fractionate mRNPs from mouse brain tissue (Han et al., 2012; Kato et al., 2012). mRNAs that precipitated with b-isox had on average 5-fold longer 3′UTRs compared to mRNAs recovered in the soluble fraction. Moreover, precipitated mRNAs encoded roughly 10-fold more binding sites for Pumilio proteins. This further suggests that 3′UTRs and their ability to bind multiple RBPs play an important role in mRNP assembly.

Originally named GW bodies because they contained an important fraction of the miRISC component GW182, P-bodies (for processing bodies) were later renamed because they also co-localized with decapping and decay proteins (Eystathioy et al., 2002, 2003). Because of this association, P-bodies have long been suspected to be sites of mRNA degradation (Sheth and Parker, 2003). They were also proposed as the site for RNAi, and several other mRNA decay activities (Unterholzner and Izaurralde, 2004; Jakymiw et al., 2005; Liu et al., 2005; Sheth and Parker, 2006). These functions, however, had been inferred and not directly demonstrated, and several studies challenged this role for P-bodies over the years (Chu and Rana, 2006; Eulalio et al., 2007b). Early on, a study by Izaurralde’s group revealed that while miRNA-mediated silencing promoted P-body formation, detectable P-bodies were not required for miRNA function (Eulalio et al., 2007b). More recently, the Weil group developed a FACS-based method to purify endogenous P-bodies and sequenced their RNA contents. With this method, they could not detect any mRNA decay intermediates (Hubstenberger et al., 2017). Interestingly, they also found that mRNAs in P-bodies were translationally repressed. They thus proposed that mRNP formation may increase the local concentration of translational repressors and thus maintain mRNA targets in a translationally repressed state. Similarly, another group monitored the dynamics of XRN1 (which mediates the 5′- > 3′ activity in many mRNA decay pathways) using an elegant dual fluorescent reporter design. Surprisingly, they noted that mRNA decay occurred throughout the cytoplasm, but not in P-bodies. This led them to also suggest that P-bodies are sites for mRNA storage, and not decay (Horvathova et al., 2017). This model nonetheless remains at striking odds with the localized concentration of decapping and decay enzymes in P-bodies.

Part of the solution to this conundrum may come from examining the composition and properties of P-bodies in different cellular lineages. The Seydoux group showed that the biochemical composition of P-bodies matured during early embryonic development, as it gained important decapping cofactors (Gallo et al., 2008). This stands to reason considering the dependence of mRNPs on the composition and concentration of proteins and mRNAs that are present in a particular context. P-bodies may have very different properties and functions in lineages as distinct as a neuron, an oocyte, an early blastomere, or an epithelial cell.

The properties of the proteins that are recruited to a 3′UTR target of miRISC or an RBP may also influence mRNP structure and activities. A recent study in *C. elegans* embryos suggested that recruitment of the CCR4-NOT complex and the associated IDR proteins by miRISC could nucleate mRNP assembly on target mRNAs. Recruitment of cell-lineage specified IDR proteins (such as PGL-1 or MEG-1/2) or co-factors of decapping and decay may enable progression into larger mRNP and toward context-dependent functions (Wu et al., 2017). In keeping with the importance of cellular context, a recent study by the Simard lab showed that miRISC has a distinct composition in *C. elegans* germline. While germline miRNA target reporters were silenced, single-molecule FISH methods revealed that targeting led to juxtaposition to P granules (germ granules) and also stabilized the targeted mRNA (Dallaire et al., 2018).

Lastly, a recent intriguing study showed that interactions between GW182 and the Argonaute could result in formation of miRISC droplets. This phase-separated condensate could in turn lead to sequestration of miRNA targets, and acceleration of their deadenylation *in vitro* (Sheu-Gruttadauria and MacRae, 2018). It thus seems likely that resolving the functions of P-bodies will be undissociable from the cellular expression and the sub-cellular concentration of mRNAs, IDRs, regulatory factors and effector machineries. Advances in quantitative methods to locally trace translation, mRNA deadenylation and decay *in situ* and in individual cell lineages may be important to resolve the apparent conflict that exists on the function of P-bodies.

## Current Frontiers in 3′UTR Research

Great strides have been made in understanding the mechanisms underlying 3′UTR regulatory sequences and the factors that recognize and effect them. However, several dating problems remain unsolved and important new ones recently emerged. The above-mentioned resolution of the functions of phase transition mRNPs provides an example of an old problem that was recently visited with a new perspective. Other important problems came into focus with the emergence of next-generation sequencing, including alternative cleavage and polyadenylation (APA) (Edwalds-Gilbert et al., 1997), which generates significant diversity in 3′UTR isoforms. High-throughput sequencing identified multiple APA sites in at least 70% of known mammalian genes (Derti et al., 2012; Hoque et al., 2012). Most tissue-specific genes express single UTRs, but more than half of ubiquitously expressed genes are produced as multiple 3′UTR isoforms (Lianoglou et al., 2013). A different choice of polyadenylation sites in a 3′UTR has the potential to profoundly re-shape its structure and response elements, thus impacting mRNA stability, translation and localization. An interesting recent study even showed that an mRNA APA can alter the localization and expression of the membrane protein it encodes (Berkovits and Mayr, 2015). Not only is there an important diversity of 3′UTR isoforms, they are also dynamic in different cellular states. On average, proliferating cells (including several tumor-derived cell lines) express shorter 3′UTRs in mRNAs that are more stable and translated into more protein compared to the longer 3′UTR mRNAs expressed in differentiated cells (Sandberg et al., 2008; Ji et al., 2009; Mayr and Bartel, 2009). This led to the idea that shorter 3′UTR isoforms allowed mRNAs to avoid regulation by miRNAs and RBPs. This is likely an over-simplification and is not always the case, however, as shorter 3′UTRs can also mean more potent deadenylation (Flamand et al., 2016), and longer 3′UTRs can also mean regulatory sequences being buried in a more complex structure (Thivierge et al., 2018). Furthermore, some tissues like the brain (Ji et al., 2009; Hilgers et al., 2011; Ulitsky et al., 2012; Miura et al., 2013) have on average much longer 3′UTRs, potentially multiplying the folding structures and/or regulatory input, and thus the complexity of functional interplay.

The folding structures of 3′UTRs remain largely under-appreciated. This in itself is an important frontier, as structures can profoundly impact gene regulation (for reviews, see Jacobs et al., 2012; Kwok et al., 2015). Significant advances in chemical probes and next-generation sequencing now enable us to obtain genome-wide *in vivo* structures at single nucleotide resolution (Bevilacqua et al., 2016). Structures can be derived from *in vivo* transcripts, thus providing a perspective on the impact of developmental and cellular contexts, and the prevailing 3′UTR interactions (Spitale et al., 2015). Along those lines, a recent study analyzed changes in structures in zebrafish transcripts during MZT (Beaudoin et al., 2018), and revealed the interplay between ribosomes and the unwinding of mRNA secondary structures.

Improvements in throughput, library generation methods, and cost-effectiveness of next-generation sequencing now enable an integrated genomic perspective on multiple regulatory mechanisms. Massively parallel reporter assays (MPRA) have been used in the past to identify functional *cis*-regulatory elements in transcription and splicing (Melnikov et al., 2012; Rosenberg et al., 2015). Thousands of random sequences with unique tags are fused to reporters and introduced into cells, and their regulatory output is then quantified using high-throughput sequencing. A recent study used a similar technique to identify *cis*-regulatory elements in the 3′UTRs of maternal mRNAs in zebrafish that regulate mRNA decay (Rabani et al., 2017). The authors identified 2 stabilizing elements (polyU and UUAG sequences) and four destabilizing elements (GC- rich, AU-rich, Pumilio-binding sites, and miR-430-binding sequences).

Because so many mechanisms mobilize the deadenylase complex and its activities, sequencing libraries that allow the capture of poly(A) tail size, the end of the 3′UTR isoform, and the abundance of transcripts will provide insight on the impact of these key features on gene expression. Recent studies already identified distinct populations of poly(A) tail sizes in the transcriptome (Subtelny et al., 2014; Eichhorn et al., 2016; Lima et al., 2017).

## Conclusion

We have reviewed how regulatory elements in 3′UTRs are recognized by miRNAs and RBPs, and some of the better-known mechanisms leading to the decisions on the fate of mRNAs. While genomic approaches are successful in unveiling the complexity and breadth of some of these mechanisms, each 3′UTR is also unique and has co-evolved closely in its genetic and cellular niche with its regulatory factors. Deciphering the 3′UTR code will also require detailing this uniqueness for each 3′UTR. Embracing genetics once more, this time through genome edition in model organisms, offers powerful new possibilities in linking the structures and sequences of 3′UTRs with mRNA fates in their physiological context.

## Author Contributions

VM and TD contributed equally in writing the manuscript.

## Conflict of Interest Statement

The authors declare that the research was conducted in the absence of any commercial or financial relationships that could be construed as a potential conflict of interest.
